# Gender Differences in Treatment-Seeking Behavior and Outcomes in Generalized Anxiety Disorder: A Retrospective Study

**DOI:** 10.7759/cureus.83872

**Published:** 2025-05-10

**Authors:** Abdulkarim O Alanazi, Abdulmajeed A Alghamdi, Rania Alessa, Mohammad N Alsalem, Nawal Alotaibi

**Affiliations:** 1 Psychiatry Department, Prince Sultan Military Medical City, Riyadh, SAU; 2 Psychiatry, King Fahad Medical City, Riyadh, SAU

**Keywords:** gad, gender differences, generalized anxiety disorder, help-seeking behavior, psychotherapy

## Abstract

Background and objective

The Diagnostic and Statistical Manual of Mental Disorders, Fifth Edition (DSM-5) defines generalized anxiety disorder (GAD) as an anxiety disorder characterized by excessive anxiety and worry occurring more days than not for at least six months, about several events or activities. Despite substantial knowledge about its treatment, limited evidence exists on gender-related differences in treatment-seeking behavior and outcomes. Understanding these differences is particularly crucial in Saudi Arabia, where the mental health system is undergoing transformation under Vision 2030. Hence, this study aimed to analyze gender-related differences in treatment-seeking behaviors, treatment preferences, adherence, functional improvement, and recurrence patterns among individuals diagnosed with GAD

Methods

A retrospective observational study was conducted using clinical records of 151 GAD patients at Prince Sultan Military Medical City (PSMMC) in Riyadh. The data, including demographic information, treatment modalities, adherence, outcomes, and recurrence, were extracted from electronic health records collected over two years since the inception of the electronic system. Statistical analyses were performed using SPSS Statistics version 26 (IBM Corp., Armonk, NY), with significance at p<0.05.

Results

The population included 64.9% females and 35.1% males, with a mean age of 45.7 years. Female patients were more likely to receive combination therapy (p=0.006) and showed significantly higher treatment effectiveness (p=0.011) and functional improvement (p=0.003). Male patients had a longer duration of symptoms before seeking treatment (p=0.022) and were more likely to receive pharmacotherapy alone. No significant differences between genders were found in the rate of recurrence or relapse.

Conclusions

Gender exerts a considerable influence on the management strategies and outcomes associated with GAD. Female patients tend to benefit more from combined treatment modalities, whereas male patients often delay treatment and prefer pharmacotherapy alone. These findings underscore the need for gender-sensitive mental health interventions to improve treatment efficacy and accessibility for all individuals with GAD.

## Introduction

According to the Diagnostic and Statistical Manual of Mental Disorders, Fifth Edition (DSM-5), generalized anxiety disorder (GAD) is classified as an anxiety disorder characterized by excessive anxiety and worry occurring more days than not for at least six months, about several events or activities [1]. Given its chronicity and pervasive nature, it is considered a significant clinical issue, and it can affect everyday functioning and quality of life. GAD is often treated with a mix of medication and psychotherapy, which is tailored to the requirements and preferences of the patient [2].

There is limited evidence in the literature about the role of gender in treatment-seeking behaviors and outcomes, despite our understanding of GAD and its management. However, women are more likely than men to suffer from anxiety disorders, as indicated by epidemiological data, suggesting possible differences in symptomatology as well as in patterns of behavior related to seeking help and treatment results [3]. To guarantee the delivery of efficient and equitable treatment, a thorough assessment of how gender affects the course of GAD management is critical.

The decision to pursue professional help for mental illnesses might be influenced by complex cultural and social factors such as societal expectations of masculinity and femininity [4]. Consequently, there are notable gender-related differences in access to mental healthcare [4]. Because seeking mental healthcare might go against social standards that emphasize emotional control, independence, and self-reliance as essential components of masculinity, males are more affected by gendered role conflicts [4]. In Saudi Arabia, where the mental healthcare system is presently undergoing significant transformation through the implementation of a new patient-centered model aligned with Vision 2030 goals, gender-based disparities highlight the significance of comprehending gender dynamics in mental health management [5]. This transformation involves integrating mental healthcare into primary healthcare settings, increasing community awareness, and addressing existing gaps to enhance service accessibility for both men and women [5].

This study aimed to investigate gender-related differences in treatment-seeking behaviors, treatment preferences (pharmacotherapy, psychotherapy, or combined modalities), adherence, functional improvement, and recurrence patterns among individuals diagnosed with GAD. Through a retrospective observational analysis of clinical data collected over two years, this research seeks to highlight gender-specific dynamics to inform and enhance gender-sensitive mental health interventions and treatment efficacy.

## Materials and methods

Study design

This study involved a single-center, observational, retrospective, cross-sectional research, based on an electronic database to examine the influence of gender on treatment-seeking behaviors, preference, and treatment outcomes among individuals diagnosed with GAD.

Data collection

Data were collected from medical records, treatment histories, and self-reported assessments of individuals diagnosed with GAD. All diagnoses of GAD were based on clinical psychiatric evaluation using DSM-5 criteria. No structured diagnostic interviews or standardized assessment tools were employed. Data were collected from the Mental Health Clinics at Prince Sultan Military Medical City (PSMMC) in Riyadh, Saudi Arabia. The study period spanned from the implementation of the electronic health record system in September 2021 up to the initiation of data collection in June 2024.

Participant selection

The sample involved a diverse cohort of individuals diagnosed with GAD. There were no eligibility restrictions in terms of age, gender, or socioeconomic background. Stratification by gender would ensure balanced representation. Participants were selected consecutively based on the availability of complete medical records that met the inclusion criteria during the study period. Due to the retrospective nature of the study, there was no randomization. Cases with missing key outcome data (e.g., recurrence, effectiveness) were excluded from those specific analyses. No statistical adjustments were made for potential confounding variables such as age, symptom duration, or prior treatment history.

Participants were excluded if they had any current or past psychiatric comorbidities, including but not limited to major depressive disorder, bipolar disorder, psychotic disorders, post-traumatic stress disorder, or substance use disorders. Additionally, individuals with significant neurological conditions, intellectual disability, or unstable medical illnesses were not eligible for inclusion. The authors applied these criteria to ensure the study focused specifically on individuals with a primary diagnosis of GAD without confounding clinical presentations.

Ethical consideration

This study was conducted in accordance with the ethical principles outlined in the Declaration of Helsinki and was approved by the Institutional Review Board (IRB) at PSMMC in Riyadh, Saudi Arabia (approval no. E-2200, dated 19/10/2023). Given the retrospective and anonymized nature of the data, informed consent was waived by the IRB. All patient data were handled with strict confidentiality and securely extracted from the hospital’s electronic medical record system. No identifiable personal information was disclosed at any stage of the study to ensure the privacy and rights of the individuals involved were fully protected.

Statistical analysis

The mean and standard deviation (SD) were used for the descriptive analysis of metric variables, while frequency and proportion (%) were employed for categorical variables. Gender was compared with the demographic and clinical characteristics of the patients by using the Chi-square test or Fisher's exact test. Values were considered significant at the p<0.05 level. All analyses were performed using the software program SPSS Statistics version 26 (IBM Corp., Armonk, NY).

## Results

This study analyzed 151 patients, with a mean age of 45.7 (SD: 19.6) years. Of them, 50.3% were older than 45 years. Females (64.9%) significantly outnumbered male patients (35.1%); 38.4% were suffering from anxiety for one to three years. Most of the patients were taking medication (64.9%). Among patients who received psychotherapy (N=53), 71.7% received at least 1-10 sessions. The most common reason for not completing sessions was “missed appointment.” The effectiveness of treatment was seen in nearly 70% of the patients, and 72.2% demonstrated functional improvement. Overall, 37.7% reported experiencing recurrence (Table 1).

**Table 1:** Demographic and clinical characteristics of the patients (n=151) SD: standard deviation

Variables	Values
Age, years, mean ± SD	45.7 ± 19.6
≤45 years, n (%)	75 (49.7%)
>45 years, n (%)	76 (50.3%)
Gender, n (%)	
Male	53 (35%)
Female	98 (65%)
Symptom duration, n (%)	
Less than 1 year	35 (23%)
1-3 years	58 (38%)
4-5 years	34 (22.5%)
More than 5 years	24 (16%)
Assessment of treatment preferences, n (%)	
Medication	98 (65%)
Psychotherapy	07 (05%)
Combination	46 (30.5%)
Number of psychotherapy sessions (n=53), n (%)	
1-10 sessions	38 (72%)
11-20 sessions	13 (24.5%)
More than 20 sessions	02 (04%)
Reason for not completing (n=53), n (%)	
Not specified	09 (17.0%)
·Ongoing	09 (17.0%)
No reason	17 (32%)
Missed appointment	12 (23%)
Others	06 (11%)
Treatment effectiveness, n (%)	
Not specified	05 (03%)
Effective	105 (69.5%)
Partially effective	29 (19%)
Not effective	08 (05%)
Lost to follow-up	04 (03%)
Functional Improvement, n (%)	
Not specified	07 (05%)
No improvement	10 (07%)
Partial improvement	25 (17%)
Improved	109 (72%)
Recurrence and relapse, n (%)	
Not specified	81 (53.6%)
Recurrence	57 (378%)
Relapse	13 (09%)

We assessed gender differences in relation to the demographic and clinical characteristics of the patients and found that male patients tended to have a longer duration of anxiety (X^2^=9.649; p=0.022), while female patients were associated with taking combined therapy (X^2^=9.977; p=0.006); females also reported higher treatment effectiveness (X^2^=8.782; p=0.011) and had better functional improvement (X^2^=11.322; p=0.003) than male patients. No gender-related differences were observed in terms of age, number of psychotherapy sessions, reason for not completing, and recurrence or relapse (p>0.05) (Table 2).

**Table 2:** Association between gender and demographic and clinical characteristics of the patients (n=151) ^§^P-value has been calculated using the Chi-square test. ^‡^P-value has been calculated using Fisher's exact test. ^*^Significant at p<0.05 level

Variables	Male, n (%) (n=53)	Female, n (%) (n=98)	X^2^	P-value^§^
Age in years				
≤45	30 (57%)	45 (46%)	1.571	0.210
>45	23 (43%)	53 (54%)		
Symptom duration				
Less than 1 year	11 (21%)	24 (24.5%)	9.649	0.022^*^
1-3 years	18 (34%)	40 (41%)		
4-5 years	09 (17%)	25 (25.5%)		
More than 5 years	15 (28%)	09 (09%)		
Assessment of treatment preferences^‡^				
Medication	39 (74%)	59 (60%)	9.977	0.006^**^
Psychotherapy	05 (09%)	02 (02%)		
Combination	09 (17%)	37 (38%)		
Number of psychotherapy sessions (n=53)^‡^				
≤10 sessions	11 (79%)	27 (69%)	0.443	0.732
>10 sessions	03 (21%)	12 (31%)		
Reason for not completing (n=53)^‡^				
No reason	04 (40%)	12 (50%)	1.488	0.542
Missed appointment	03 (30%)	09 (37.5%)		
Others	03 (30%)	03 (12.5%)		
Treatment effectiveness^‡^				
Effective	29 (59%)	76 (82%)	8.782	0.011^*^
Partially effective	15 (31%)	14 (15%)		
Not effective	05 (10%)	03 (03%)		
Functional improvement				
No improvement	05 (10%)	05 (05%)	11.322	0.003^**^
Partial improvement	15 (31%)	10 (10.5%)		
Improved	29 (59%)	80 (84%)		
Recurrence and relapse^‡^				
Recurrence	22 (85%)	35 (79.5%)	0.278	0.754
Relapse	04 (15%)	09 (20.5%)		

## Discussion

Our findings highlight notable gender differences in the management and treatment outcomes of GAD. In contrast to male patients, females were more likely to receive a combination of pharmacotherapy and psychotherapy, showing higher treatment effectiveness and better functional improvement. Conversely, male patients had a longer duration of untreated anxiety before seeking treatment. Although the study found that female patients were more likely to receive combination therapy and demonstrated better outcomes, it is important to consider that this treatment approach may also be indicative of greater clinical severity or complexity. In routine practice, clinicians often select combined pharmacotherapy and psychotherapy for patients who present with more persistent or severe symptoms. However, in our dataset, there was no standardized or consistently documented information regarding symptom severity or the rationale behind treatment selection. As such, the observed association between combination therapy and improved outcomes in female patients should be interpreted with caution, as it may reflect clinical decision-making based on unmeasured severity rather than inherent treatment preference or efficacy.

A notable disparity was observed in treatment preferences, with female patients more likely to undergo combined therapy (p=0.006). This finding is consistent with Addis et al., who reported that men are generally less likely than women to seek psychiatric services, psychotherapy, and counseling, which could explain the higher preference for combined therapy among women [6]. Moreover, Otto et al. examined the effectiveness of combined therapy and found that while pharmacotherapy and cognitive-behavioral therapy (CBT) are both effective in treating anxiety disorders, combined therapy does not always yield superior results compared to monotherapy, suggesting that gender-specific preferences may influence treatment outcomes [7]. Conversely, male patients in the present study demonstrated a marked preference for pharmacotherapy alone (73.6% vs. 60.2% in females), which is consistent with results presented in previous studies showing that men are less likely to engage in psychotherapy due to beliefs surrounding masculinity and social stigma [6]. Addis et al. explained that traditional masculine norms discourage men from expressing emotional vulnerability, leading to reduced engagement with psychotherapy [6]. This suggests a need for targeted interventions to encourage male patients to consider psychotherapy as a viable treatment option.

In contrast to male patients, female patients had significantly higher treatment effectiveness (p=0.011) and better functional improvement (p=0.003). Social support networks, which are often stronger among women, may contribute to this enhanced treatment effectiveness [8]. Furthermore, Cuijpers et al. found that psychotherapy, particularly CBT, is highly effective in reducing GAD symptoms and improving quality of life [9]. The greater willingness of female patients to participate in psychotherapy sessions may explain their superior functional outcomes observed in this study.

Another notable observation was that male patients had a significantly longer duration of untreated anxiety before seeking treatment (p=0.022). Addis et al. found that men, compared to women, were more likely to delay seeking professional help for mental health conditions [6]. This delay in treatment can exacerbate symptoms and lead to chronicity, which underscores the importance of early intervention strategies specifically targeting male populations. Furthermore, Kohn et al. highlighted that despite the availability of effective treatments, due to several factors such as social stigma, lack of awareness, and perceived inefficacy of therapy, a significant portion of individuals with mental illnesses remain untreated [10]. This is especially relevant in Saudi Arabia, where cultural norms and a lack of mental health literacy may make it more difficult for patients, particularly men, to receive prompt therapy [11]. Addressing these barriers could improve early treatment engagement, particularly among male patients.

No statistically significant differences were found in recurrence or relapse rates between male and female patients (p>0.05), although there were gender differences in treatment effectiveness and functional improvement. In contrast to some clinical studies suggesting gender differences in the onset and course of anxiety disorders, epidemiological research, including findings by McLean et al., indicates that men and women follow a similar developmental trajectory in terms of anxiety disorder onset, though women exhibit a higher overall risk throughout their lifespan [12]. This suggests that while gender may influence prevalence rates, the persistence and progression of anxiety disorders are likely shaped more by individual symptom severity and comorbidities rather than gender alone.

This study is limited by its retrospective design, absence of standardized outcome measures, and reliance on subjective clinical documentation. Additionally, high rates of missing data in recurrence variables and the lack of control for confounding factors limit the generalizability of findings. The findings of this study highlight the critical need for employing gender-sensitive approaches in the management of GAD. Encouraging male patients to engage in psychotherapy, addressing stigma-related barriers, and offering tailored interventions may improve outcomes. Moreover, it is imperative to underscore the significance of prolonged follow-up and support for individuals of both genders to improve adherence to treatment protocols and diminish the probability of relapse occurrences. Future research should explore additional factors, such as cultural influences and socioeconomic status, contributing to gender differences in mental illness management. Qualitative studies examining patients’ perspectives on treatment choices and adherence barriers could provide deeper insights into optimizing mental health interventions.

## Conclusions

Our findings contribute to the existing body of literature regarding gender differences in GAD treatment, highlighting that female patients tend to participate more in combined pharmacotherapy and psychotherapy, resulting in better functional outcomes. In contrast, male patients experienced longer durations of untreated anxiety and preferred pharmacotherapy alone. These findings reinforce the necessity of gender-sensitive mental health policies and intervention strategies to ensure equitable and effective treatment for all individuals with GAD.
